# The Effect of a Nursing Hold Team on Patient Satisfaction for Admitted Patients Discharged Directly From the Emergency Department

**DOI:** 10.7759/cureus.17100

**Published:** 2021-08-11

**Authors:** Brittany Choe, Joseph Basile, Bartholomew Cambria, Elias Youssef, Mikhail Podlog, Kurien Mathews, Nicole Berwald, Barry Hahn

**Affiliations:** 1 Emergency Medicine, Staten Island University Hospital (SIUH), Staten Island, USA

**Keywords:** patient satisfaction, ed boarding, ed holds, press ganey, nursing hold team

## Abstract

Objectives: Emergency departments (ED) across the United States face challenges related to patient volume, available capacity, and patient throughput. Patient satisfaction is adversely affected by crowding and lengthy boarding times. This study aimed to determine whether the implementation of a dedicated nursing hold team (NHT) would improve patient satisfaction scores for admitted patients discharged directly from the ED.

Methods: This was a retrospective, observational study with a pre-/post-test design. All admitted adult patients who returned a Press Ganey (PG) survey were included in the study. There were two twelve-month study periods before and after implementing an ED NHT. The primary outcome was the percentage of patients who gave top box scores for all questions in the Nursing Communication Domain.

Results: During the pre-implementation period, 108 patients (59%) gave an overall top box rating for the Nursing Communication Domain versus the post-implementation period, where 99 patients (66%) provided a top box rating (OR 1.375, p = 0.16). There was a trend toward increased satisfaction for individual categories. However, these differences were not statistically significant.

Conclusions: Implementing a dedicated NHT showed an increase in the overall top box PG Nursing Communication Domain score and several of the individual domain questions. Future studies should examine other potential benefits from a dedicated NHT, such as the rate of adverse events and medication delays.

## Introduction

Emergency departments (ED) across the United States face daily challenges related to patient volume, available capacity, and overall patient throughput. One of the significant contributors to these challenges includes admitted patients boarding in the ED. Holds or “boarders” refer to patients admitted to the hospital but who remain in the ED due to a lack of available inpatient beds. ACEP defines a “boarded patient” as a patient who remains in the ED after the patient has been admitted or placed into observation status at the facility, but has not been transferred to an inpatient or observation unit. This cohort introduces many logistical and staffing constraints that must be navigated daily. Primarily, boarders compete with new ED patient arrivals for nursing and patient care resources. Boarders also have higher rates of medication delays and adverse events [1-3]. Furthermore, both treat and release and admitted patient satisfaction is adversely affected by ED crowding, prolonged ED hallway time, and lengthy boarding times [4-5].

The impact of nursing workflow and staffing patterns on overall patient satisfaction has been well documented in the literature [6-8]. Studies simulating inpatient experiences have demonstrated that boarding patients negatively impacts patient satisfaction and patient outcomes [9-10]. A study by Chadaga et al. showed that utilizing a hospital medicine ED team can improve patient flow, provide more timely care, and improve patient satisfaction to patients boarding in the ED. The Staten Island University Hospital currently employs such a strategy [9]. To further mitigate the staffing constraints and competing patient care priorities imposed by the holds, the ED was provided with a dedicated nursing hold team (NHT). Prior to this implementation, ED nurses continued to care for patients even after they were admitted. After the implementation of a NHT, when a patient was admitted, their care would be transferred to the NHT. This change allowed ED nurses to be available to attend to new ED arrivals. Boarded patients were provided the same level of nursing care administered on the inpatient units. The goal of this study was to determine whether the implementation of an NHT would improve patient satisfaction scores for admitted patients discharged directly from the ED.

## Materials and methods

This was a retrospective, observational study with a pre-/post-test design. The study included two 12-month study periods approximately one year apart. The pre-implementation period used for comparison was November 1, 2017 - October 31, 2018, and the post-implementation period was November 1, 2018 - October 31, 2019. All admitted patients who returned a Press Ganey (PG) survey older than 21 years of age were included in the study. Patients and providers were identified through the Press Ganey database.

The study was conducted at Staten Island University Hospital, a 700-bed tertiary-care teaching hospital in Staten Island, NY. The ED has a census of approximately 97,000 patient visits per year. The ED is comprised of 75 patient care areas that can be flexed up to 120 care areas. On average, holds account for approximately 25 of these slots daily. It is not uncommon to hold more than 40 patients during periods of high volume. The local institutional review board approved this study.

The intervention was the implementation of an NHT. This team comprised four nurses: three general ED nurses and one critical-intensive care unit (ICU) nurse. The general hold nurses could each care for a maximum of seven patients. An ICU hold nurse could care for two ICU holds and be repurposed to the general ED hold team during low critical care periods. The hold nurses all received their training in the ED and inpatient units and were comfortable with the care required for an admitted patient. The daily availability of a fourth ED hold nurse was dependent on the number of ED holds an overall RN availability based on staffing. However, there was always a minimum of three ED hold nurses 24/7. Additionally, three dedicated Patient Care Assistants (PCAs) were assigned to the NHT as part of this team. PCAs were responsible for assisting with patient care needs such as hygiene and bedding, repeating vital signs, and performing one-to-one patient observations.

The PG survey (Press Ganey Associates, Inc., South Bend, IN) is a patient experience survey mailed to patients upon discharge from the ED or the hospital after admission. It is a nationally recognized survey commonly used to measure a patient’s perception of healthcare delivery. The PG survey uses a Likert-type scale of 5 responses: very poor, poor, fair, good, and very good.

**Figure 1:**
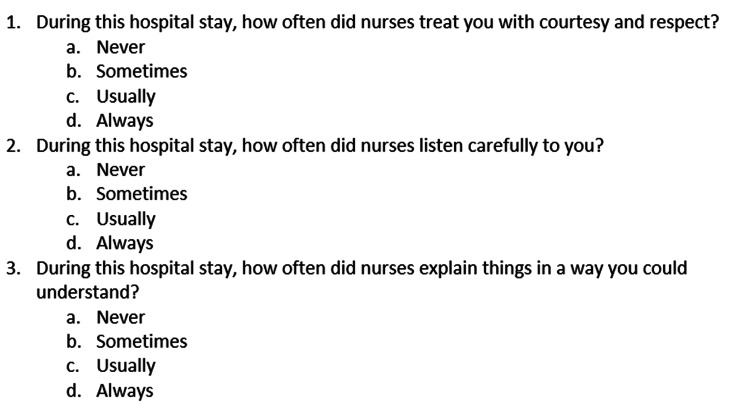
Press Ganey Questions

This study’s primary outcome was the percentage of patients who gave top box scores for all of the Nursing Communication Domain questions of hold patients. The percentage of responses answered “very good” is called the Top Box score. The Top Box score is used by PG and health-care institutions to make comparisons between locations, individual, and entire health-care institutions. The percentage of top box scores for three of the domain questions was also analyzed individually. The Nursing Communication Domain questions look directly at the care provided by a patient’s nurse and are most representative of the impact of the NHT. Top box scores indicate when a patient answered the highest response possible on the survey scale (e.g., percentage of “Always” or “Very Good”). PG metrics are typically calculated in terms of top box scores, and comparative PG data between hospitals is evaluated in terms of top box scores. All questions used in the study are listed in Figure 1.

We analyzed the PG scores of all patients who were admitted to the hospital and were discharged directly from the ED. Patients who are discharged from the ED receive a PG survey asking about their ED experience. While patents who are admitted to an inpatient floor receive a separate survey asking about their inaptient experience. To capture patient satisfaction as it related to the NHT, patients cared for by the NHT and later moved to the inpatient floors were not included in the study. These patients were excluded to ensure that the PG scores were directly related to the care provided by the NHT and not to the inpatient nurses caring for the patient. By analyzing the PG scores of admitted patients discharged directly from the ED before and after implementing the NHT, we assessed the effect of added resources and dedicated nursing care on overall patient satisfaction.

The data was collected and managed using REDCap, a secure, web-based application designed to support data capture for research studies. No sample size was calculated as the whole eligible population during the designated study periods were included in the study. Our data was analyzed in terms of the percentage of top box scores. Top box scores were summarized with frequency counts and percentages. Comparisons of top box scores between pre- and post-implementation groups were evaluated using chi-square tests for binary variables. All statistical tests are two-sided, and a P-value of <0.05 is considered to indicate statistical significance. Data analyses was conducted using the Analyse-it version 4.95.4 (Analyse-it Software, Leeds, UK). 

## Results

**Figure 2:**
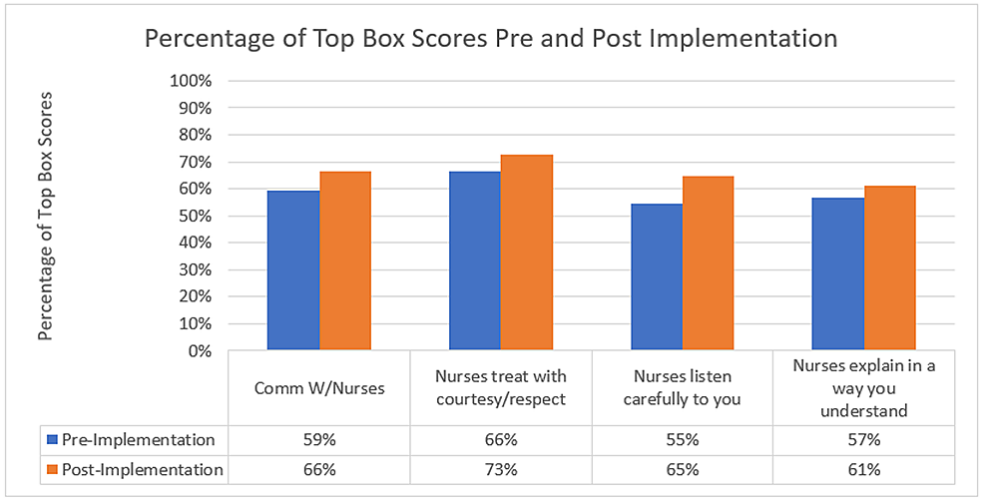
Percentage of Top Box Scores Pre and Post Implementation

During the pre-implementation period, 2714 subjects were identified as holds. 182 subjects from this group were discharged directly from the ED and subsequently completed a PG survey. During the post-implementation period, 2791 subjects were identified as holds. 149 subjects from this group were discharged directly from the ED and subsequently completed a PG survey. All subjects were included in the final analysis. Data is presented in Figure 2. During the pre-implementation period, 108 patients (59%) gave an overall top box rating for the Nursing Communication Domain versus the post-implementation period, where 99 patients (66%) provided a top box rating (OR 1.375, p = 0.16).

There was a trend toward increased satisfaction for individual categories. During the pre- and post-implementation study periods, 107 (73%) versus 120 (66%) patients reported increased satisfaction when asked if their nurses treated them with courtesy and respect (OR 1.36, p = 0.23), 95 (65%) versus 100 (55%) if nurses listened carefully (OR, 1.52, p = 0.07), and 91 (61%) versus 103 (57%) if nurses explained matters in a way that could be understood (OR 1.22, p = 0.43). However, these differences were not statistically significant.

## Discussion

The goal of this study was to determine whether the implementation of an NHT would improve patient satisfaction scores for admitted patients discharged directly from the ED. The current study observed a trend towards an increased percentage of top box PG scores for all patient satisfaction questions. However, this trend was not statistically significant in any individual questions or the Nursing Communication Domain. There is no previous literature on the implementation of an NHT and its effect on patient satisfaction. Still, studies have shown that dedicated nursing resources towards boarded patients can improve hospital workflow, reduce hospital length of stay, and improve patient walkout rates [6,8].

Several factors may account for the results of this study. Despite the addition of NHT, other factors may have negatively impacted the patient’s perception of boarding in the ED. First, the hospital medicine ED team, which manages hold patients is not physically in the ED at all times. This structure can lead to delays which patients may perceive as a lack of communication. Previous studies have demonstrated that there are often difficulties in communication between nursing and inpatient teams for boarded patients [11-14]. Another possibility regarding the lack of impact of an NHT on patients' perception of boarding in the ED is that certain inpatient comfort strategies are not feasible in the ED. McGowan et al. established that being placed in inpatient hallways is preferred over boarding in the ED for these reasons [14-20]. These factors may have negatively impacted the patient’s overall experience and their ED nursing care perception. Finally, there was a trend toward increased satisfaction with the NHT. This study was underpowered to detect statistical significance. Our findings might have been different if the study was continued for a more extended period with more subjects.

While this study's outcomes were focused on patient satisfaction, there are other potential benefits from a dedicated NHT that should be examined in the future. Boarded patients have higher rates of adverse events and medication delays, and NHTs may prevent or mitigate this outcome [1-3]. Our study also did not consider the perception of active ED patients. With the implementation of the NHT, nursing ratios for active ED patients decreased, and nursing staff was able to focus more directly on their ED patients rather than switching tasks between boarding patients and active patients. Another critical element to consider is nursing staff satisfaction. With lower nursing-to-patient ratios and less task switching between active ED and boarding patients, our nursing staff reported they could more effectively and comfortably care for patients.

There are several limitations to our study. This study was designed as a retrospective cohort study and is subject to a retrospective study's inherent bias. This study was also performed at a single-site academic center. Because we obtained data through the anonymous information provided by PG, we could not collect specific demographic data regarding our patient population.

There are many factors that could potentially affect patient satisfaction. These include diagnosis, comorbidities, daily volume, length of stay, and age. Unfortunately, it was not possible to control for all these variables. In this study, we looked at an NHT with the assumption that other variables would be evenly distributed across the sample.

A multicenter, prospective survey performed while patients were boarding in the ED could be achieved in future studies to minimize recall bias and non-response bias from PG surveys. Studies looking at other potential benefits from a dedicated NHT should also be examined in the future.

## Conclusions

Implementing a dedicated NHT showed an increase in the percentage of the overall top box PG Nursing Communication Domain score and several of the individual domain questions. Future studies should examine other potential benefits from a dedicated NHT, such as the rate of adverse events and medication delays.
